# Associations of Carotid Intima‐Media Thickness and Plaque Heterogeneity With the Risks of Stroke Subtypes and Coronary Artery Disease in the Japanese General Population: The Circulatory Risk in Communities Study

**DOI:** 10.1161/JAHA.120.017020

**Published:** 2020-09-29

**Authors:** Saeko Shimoda, Akihiko Kitamura, Hironori Imano, Renzhe Cui, Isao Muraki, Kazumasa Yamagishi, Mitsumasa Umesawa, Tomoko Sankai, Mina Hayama‐Terada, Yasuhiko Kubota, Yuji Shimizu, Takeo Okada, Masahiko Kiyama, Hiroyasu Iso

**Affiliations:** ^1^ Public Health, Department of Social Medicine Osaka University Graduate School of Medicine Osaka Japan; ^2^ Research Team for Social Participation and Community Health Tokyo Metropolitan Institute of Gerontology Tokyo Japan; ^3^ Osaka Center for Cancer and Cardiovascular Disease Prevention Osaka Japan; ^4^ Department of Public Health Medicine, Faculty of Medicine, and Health Services Research and Development Center University of Tsukuba Ibaraki Japan; ^5^ Ibaraki Western Medical Center Ibaraki Japan; ^6^ Department of Public Health Dokkyo Medical University Tochigi Japan; ^7^ Department of Public Health and Nursing, Faculty of Medicine University of Tsukuba Ibaraki Japan; ^8^ Yao Public Health Center, Yao City Office Osaka Japan

**Keywords:** carotid intima‐media thickness, coronary artery disease, lacunar infarct, plaque, stroke, Cardiovascular Disease, Epidemiology

## Abstract

**Background:**

Evidence on the associations of carotid intima‐media thickness and carotid plaque characteristics with stroke subtypes and coronary artery disease risks in Asians is limited. This study investigated these associations in the Japanese general population.

**Methods and Results:**

Maximum intima‐media thicknesses of both the common carotid artery and internal carotid artery and carotid plaque characteristics were evaluated in 2943 Japanese subjects aged 40 to 75 years without history of cardiovascular disease. Subjects were followed up for a median of 15.1 years. Using a multivariate Cox proportional hazard model, we found that hazard ratios (HRs) and 95% CIs for the highest (≥1.07 mm) versus lowest (≤0.77 mm) quartiles of maximum intima‐media thicknesses of the common carotid artery were 1.97 (1.26–3.06) for total stroke, 1.52 (0.67–3.41) for hemorrhagic stroke, 2.45 (1.41–4.27) for ischemic stroke, 3.60 (1.64–7.91) for lacunar infarction, 1.53 (0.69–3.41) for nonlacunar cerebral infarction, 2.68 (1.24–5.76) for coronary artery disease, and 2.11 (1.44–3.12) for cardiovascular disease (similar results were found for maximum intima‐media thicknesses of the internal carotid artery). HRs(95% CIs) for heterogeneous plaque versus no plaque were 1.58 (1.09–2.30) for total stroke, 1.25 (0.58–2.70) for hemorrhagic stroke, 1.74 (1.13–2.67) for ischemic stroke, 1.84 (1.03–3.19) for lacunar infarction, 1.58 (0.80–3.11) for nonlacunar cerebral infarction, 2.11 (1.20–3.70) for coronary artery disease, and 1.71 (1.25–2.35) for cardiovascular disease.

**Conclusions:**

Maximum intima‐media thicknesses of the common carotid artery, maximum intima‐media thicknesses of the internal carotid artery, and heterogeneous plaque were associated with the risks of stroke, lacunar infarction, coronary artery disease, and cardiovascular disease in Asians.

**Nonstandard Abbreviations and Acronyms**
CCA: common carotid artery
CIMT: carotid intima‐media thickness
CIRCS: Circulatory Risk in Communities Study
ICA: internal carotid artery
max CCA‐IMT: maximum intima‐media thickness of common carotid artery
max ICA‐IMT: maximum intima‐media thickness of internal carotid artery

**Clinical Perspective**

**What Is New?**

- Maximum intima‐media thicknesses of both the common carotid artery and internal carotid artery were positively associated with the risk of lacunar infarction in the Asian general population.
- Maximum intima‐media thickness of the common carotid artery was positively associated with the risk of coronary artery disease in the Asian general population.
- Carotid plaque heterogeneity was positively associated with the risks of total stroke, ischemic stroke, lacunar infarction, coronary artery disease, and cardiovascular disease.

**What Are the Clinical Implications?**

- Carotid ultrasound imaging is useful for identifying patients at high risk for stroke and coronary artery disease in Asians.

Carotid intima‐media thickness (CIMT) and carotid plaque are considered as markers of atherosclerosis, and they are known to be associated with the risk of stroke.^1^, ^2^, ^3^, ^4^ However, few studies have investigated their associations with the risks of stroke subtypes. Stroke is composed of heterogeneous subtypes: hemorrhagic or ischemic stroke. Among ischemic stroke subtypes, lacunar infarction is highly common in the Japanese population, accounting for 30% to 50% of ischemic stroke cases, while ≈20% of ischemic stroke is lacunar infarction in Western countries.^5^, ^6^, ^7^ Lacunar infarction has a 20% recurrence rate and a 25% 5‐year mortality and is associated not only with movement disorder but also with cognitive impairment.^8^ Lacunar infarction can be caused primarily by arteriolosclerosis and partially by atherosclerosis at the opening of penetrating branches located in the parent cerebral artery.^9^, ^10^ Few studies have examined the associations of CIMT and carotid plaque characteristics with the risk of lacunar infarction, and the results were inconsistent.^11^, ^12^

In Western countries, CIMT and carotid plaque have been found associated with increased risk of coronary artery disease (CAD)^1^, ^13^; however, few studies have examined these associations in the Asian population. Our previous study with a 4.5‐year follow‐up showed that CIMT and heterogeneous carotid plaque were associated with the risks of total and ischemic strokes in Japanese men.^4^ In the current community‐based prospective study, we aimed to examine the associations of CIMT and carotid plaque characteristics with the risks of total stroke, stroke subtypes, CAD, and cardiovascular disease (CVD) in the Japanese population.

## Methods

The data supporting the findings of this study are available from the corresponding author upon reasonable request.

### Study Population

We conducted a cohort study using data from the CIRCS (Circulatory Risk in Communities Study). The CIRCS is an ongoing dynamic community cohort study in Japan from 1963. Details of the CIRCS design have been described elsewhere.^14^ The present study comprised subjects aged 40 to 75 years who took the annual cardiovascular risk factor surveys in four communities of the CIRCS: Ikawa town (a rural community in Akita Prefecture, northeastern Japan) between 1996 and 2000, and 2002 and 2004; the Minami‐Takayasu district in Yao City (an urban community in Osaka Prefecture, midwestern Japan) between 1997 and 2000; Noichi town (a rural community in Kochi Prefecture, southwestern Japan) between 1996 and 1999; and Kyowa town (a rural community in Ibaraki Prefecture, mideastern Japan) in 1997 and 1999.

A total of 3073 subjects (2338 men and 735 women) who participated in the cardiovascular risk factor surveys underwent ultrasound examination, including 903 from Ikawa town, 886 from Yao City, 631 from Noichi town, and 653 from Kyowa town. Subjects with a history of stroke (n=75) or CAD (n=40) were excluded from the analysis. Further, 15 subjects without data for cardiovascular risk factors (n=6) or for CIMT (n=9) were excluded. Additionally, 1 subject without data for presence of carotid plaque was excluded from the analysis of carotid plaque characteristics. Finally, 2943 subjects (2226 men and 717 women) were included in the analyses for CIMT and 2942 subjects in the analyses for carotid plaque characteristics.

Informed consent was implied by participation in cardiovascular risk factor surveys and was obtained from representatives in communities according to the guidelines of the Council for International Organizations of Medical Science,^15^ which was a common practice at that time in Japan. The study protocol was approved by the ethics committees of the Osaka Center for Cancer and Cardiovascular Disease Prevention and of Osaka University.

### Ultrasound Imaging

A single epidemiologist (A.K.) trained in carotid ultrasonography and certified from the Cardiovascular Health Study conducted ultrasonography using a high‐resolution B‐mode ultrasound imaging unit (Toshiba, Tokyo, Japan) with a 7.5 MHz annular array probe (Toshiba SMA‐736s). The protocol was based on that of the Cardiovascular Health Study,^1^, ^16^ and the details were described in our previous report.^4^ The epidemiologist performed ultrasonographic examination and evaluated the obtained images blinded to the data from cardiovascular risk factor surveys. Maximum IMT was measured for both the common carotid artery (CCA) and internal carotid artery (ICA) because the Cardiovascular Health Study adopted the maximum CIMT as a parameter with stronger association with cardiovascular risk factors than the mean CIMT.^1^ The maximum IMT of CCA (max CCA‐IMT) was defined as the greatest IMT at the near and far walls of either the right or left CCA. The maximum IMT of ICA (max ICA‐IMT) was defined in the same way. We defined carotid plaque as focal thickness of the vessel wall ≥1.5 mm in ICA, and plaque characteristics were classified qualitatively following the same classification used in the Cardiovascular Health Study.^16^, ^17^ Plaque heterogeneity was classified as either homogeneous or heterogeneous. The plaque surface characteristics were classified as smooth, mildly irregular, markedly irregular, or ulcerated. When the subject had multiple plaques, the thickest plaque was evaluated.

### Risk Factor Measurements

The protocols for the cardiovascular risk factor surveys have been described in detail in our previous report.^4^ Body mass index was calculated as weight (kg) divided by the height squared (m^2^). Blood pressure was measured by trained observers using standard mercury sphygmomanometers in a quietly seated position after a ≥5‐minute rest. We identified current smokers as those who smoked ≥1 cigarette per day, ex‐smokers as those who had quit smoking for ≥3 months, current drinkers as those who drank ≥1 times per week, and ex‐drinkers as those who had not drunk for ≥3 months according to the subjects' reports. We defined diabetes mellitus as fasting glucose ≥7.0 mmol/L, nonfasting glucose ≥11.1 mmol/L, or current use of antidiabetic drugs. Non–high‐density lipoprotein cholesterol was calculated by subtracting the high‐density lipoprotein cholesterol (mmol/L) value from the total cholesterol (mmol/L) value. Creatinine was assayed using the Jaffe method before 2001 and with an enzymatic method after 2001. Creatinine values obtained by Jaffe method were converted into those obtained by an enzymatic method and the estimated glomerular filtration rate (eGFR) was calculated using the following equation proposed by the working group of the Japanese Chronic Kidney Disease initiative^18^: eGFR (mL/min per 1.73 m^2^)=194×(creatinine [an enzymatic method])^−1.094^×(age)^−0.287^×(0.739 for women). A 12‐lead ECG was obtained, and atrial fibrillation was determined according to Minnesota codes 8‐3‐1.^19^

### Cardiovascular End Points

Subjects were followed until the end of 2017 for Ikawa, 2016 for Yao, 2009 for Noichi, and 2014 for Kyowa. Subjects were censored when they withdrew from the cohort because of deaths without prior CVD incidence (n=753) or moved out of the communities (n=103).

We defined CVD as CAD and stroke. Stroke subtypes were identified as intraparenchymal hemorrhage, subarachnoid hemorrhage, lacunar infarction, large‐artery embolism, large‐artery thrombosis, unclassified large‐artery infarction, or unclassified stroke. CAD was defined as definite or possible myocardial infarction, angina pectoris, and sudden cardiac death. The details of the criteria for diagnosis of stroke and CAD are shown in Data S1. The final diagnoses of CVD were independently determined by 2 or 3 physician‐epidemiologists who were blinded to the data from the cardiovascular risk factor surveys and ultrasound findings.

### Statistical Analysis

Baseline demographic characteristics of the subjects were summarized according to the quartiles of max CCA‐IMT or max ICA‐IMT. We reported the percentage of men and the mean value with standard deviation for age. For other values, except for age and sex, we reported age‐, sex‐, and community‐adjusted means with standard errors for continuous variables or percentages for categorical variables. We performed linear tests for trend using the median values of max CCA‐IMT or max ICA‐IMT within each quartile as ordinal variables. Cox proportional hazards regression models were used to estimate the hazard ratios (HRs) and 95% CIs for incident total stroke, hemorrhagic stroke, ischemic stroke, lacunar infarction, nonlacunar cerebral infarction, CAD, and CVD for each quartile of maximum CIMT relative to the lowest quartile of maximum CIMT. We selected adjustment variables based on previous studies.^20^, ^21^ The initial model was adjusted only for age, sex, and community. The multivariable models were further adjusted for body mass index, systolic blood pressure, use of antihypertensive medication, diabetes mellitus, non–high‐density lipoprotein cholesterol, triglycerides, use of cholesterol‐lowering medication, eGFR, atrial fibrillation, smoking status, and drinking status. The corresponding HRs and 95% CIs for plaque characteristics relative to the subjects without plaque were estimated using Cox proportional hazards regression models and the multivariable models were adjusted for the same variables as those in the models for maximum CIMT. In order to examine whether max CCA‐IMT was independently associated with cardiovascular outcomes, we further adjusted for the presence of plaque in addition to other cardiovascular risk factors. All statistical analyses were performed with the SAS for Windows (version 9.4; SAS Inc., Cary, NC). All *P* values for statistical tests were 2‐tailed, and those <0.05 were considered statistically significant.

## Results

Table 1 shows the percentage of men; the mean value with standard deviation for age; and age‐, sex‐, and community‐adjusted mean values and prevalence rates of the cardiovascular risk factors, except for age and sex, at baseline according to max CCA‐IMT or max ICA‐IMT quartiles. The mean age, body mass index, and total cholesterol, non–high‐density lipoprotein cholesterol, and triglyceride levels were higher for the higher max CCA‐IMT quartile. The prevalence of diabetes mellitus, atrial fibrillation, and current smokers was higher for the higher max CCA‐IMT quartile. The mean eGFR, high‐density lipoprotein cholesterol level, and the prevalence of current drinkers showed inverse associations with max CCA‐IMT. There was no significant trend for the prevalence of cholesterol‐lowering medication. For max ICA‐IMT quartiles, most cardiovascular risk factors had the same trends as those for max CCA‐IMT quartiles, but there were no significant trends for diastolic blood pressure, total cholesterol level, high‐density lipoprotein cholesterol level, eGFR, and the prevalence of current drinkers.

**Table 1:** Baseline Characteristics According to Quartiles of Max CCA‐IMT and Max ICA‐IMT

	Max CCA‐IMT				*P* for Trend
	≤0.77 mm	0.78–0.95 mm	0.96–1.06 mm	≥1.07 mm	
Number of participants	862	610	741	730	
Men, %	74.8	72.6	74.0	80.8	<0.001
Age, y	62.6 (6.9)	65.2 (5.8)	66.6 (5.4)	66.6 (5.6)	<0.001
Body mass index, kg/m^2^	22.7 (0.1)	23.7 (0.1)	23.6 (0.1)	24.2 (0.1)	<0.001
Systolic blood pressure, mm Hg	134.1 (0.6)	134.5 (0.7)	137. 6 (0.6)	140.7 (0.6)	<0.001
Diastolic blood pressure, mm Hg	81.8 (0.4)	81.8 (0.4)	82.3 (0.4)	82.8 (0.4)	0.04
Use of antihypertensive medication, %	23.1	23.9	30.4	34.9	<0.001
Diabetes mellitus, %	5.3	7.2	9.4	14.3	<0.001
Total cholesterol, mmol/L	5.1 (0.03)	5.2 (0.03)	5.3 (0.03)	5.5 (0.03)	<0.001
High‐density lipoprotein cholesterol, mmol/L	1.5 (0.01)	1.4 (0.02)	1.4 (0.01)	1.4 (0.01)	<0.001
Non–high‐density lipoprotein cholesterol, mmol/L	3.6 (0.03)	3.8 (0.03)	3.8 (0.03)	4.1 (0.03)	<0.001
Triglycerides, mmol/L	1.3 (0.03)	1.5 (0.03)	1.5 (0.03)	1.5 (0.03)	0.0007
Use of cholesterol‐lowering medication, %	6.9	6.5	9.2	7.9	0.33
eGFR, mL/min per 1.73 m^2^	77.6 (0.8)	76.7 (0.9)	77.6 (0.9)	73.5 (0.9)	0.0004
Atrial fibrillation, %	1.2	1.0	1.2	2.4	0.02
Current smokers, %	31.2	32.7	32.7	36.4	0.03
Current drinkers, %	57.9	58.1	55.6	52.1	0.004

	Max ICA‐IMT				*P* for Trend
	≤0.98 mm	0.99–1.25 mm	1.26–1.83 mm	≥1.84 mm	
Number of participants	735	743	758	707	
Men, %	69.3	71.7	77.4	84.4	<0.001
Age, y	63.5 (6.7)	64.1 (6.4)	65.5 (6.0)	66.9 (5.2)	<0.001
Body mass index, kg/m^2^	23.6 (0.1)	23.6 (0.1)	23.6 (0.1)	23.3 (0.1)	0.04
Systolic blood pressure, mm Hg	134.6 (0.6)	135.1 (0.6)	137.5 (0.6)	139.8 (0.7)	<0.001
Diastolic blood pressure, mm Hg	82.1 (0.4)	82.2 (0.4)	82.3 (0.4)	82.2 (0.4)	0.80
Use of antihypertensive medication, %	22.4	24.5	28.8	36.8	<0.001
Diabetes mellitus, %	7.0	7.6	8.8	12.7	<0.001
Total cholesterol, mmol/L	5.2 (0.03)	5.3 (0.03)	5.3 (0.03)	5.3 (0.03)	0.014
High‐density lipoprotein cholesterol, mmol/L	1.5 (0.01)	1.5 (0.01)	1.4 (0.01)	1.5 (0.01)	0.99
Non–high‐density lipoprotein cholesterol, mmol/L	3.7 (0.03)	3.8 (0.03)	3.9 (0.03)	3.9 (0.03)	0.009
Triglycerides, mmol/L	1.4 (0.03)	1.4 (0.03)	1.5 (0.03)	1.5 (0.03)	0.0024
Use of cholesterol‐lowering medication, %	5.6	7.0	8.0	10.0	<0.001
eGFR, mL/min per 1.73 m^2^	77.2 (0.9)	77.4 (0.9)	75.4 (0.9)	75.6 (0.9)	0.14
Atrial fibrillation, %	1.0	0.9	0.9	3.2	<0.001
Current smokers, %	28.6	29.1	35.8	39.4	<0.001
Current drinkers, %	53.7	56.3	55.9	57.7	0.14

The value for men is presented as proportion. Age is presented as mean (standard deviation). Other values are presented as mean (standard error) or proportions adjusted by age, sex, and community. eGFR indicates estimated glomerular filtration rate; max CCA‐IMT, maximum intima‐media thickness of the common carotid artery; and max ICA‐IMT, maximum intima‐media thickness of the internal carotid artery.

During the median follow‐up of 15.1 years (maximum 21.6 years), stroke occurred in 186 subjects, and the incidence rate per 1000 person‐years was 4.3. Among subjects with total stroke, 137 subjects had ischemic stroke. Lacunar infarction occurred in 77 subjects, which accounted for 41.4% of total stroke cases and 56.2% of ischemic stroke cases. CAD occurred in 88 subjects, and the incidence rate per 1000 person‐years was 1.9.

Table 2 shows HRs (95% CIs) of total stroke, stroke subtypes, CAD, and CVD according to quartiles of max CCA‐IMT. Max CCA‐IMT was significantly associated with the risks of total stroke, CAD, and CVD. When we examined the associations according to stroke subtypes, we found significant associations for ischemic stroke and lacunar infarction but not for hemorrhagic stroke and nonlacunar cerebral infarction. The multivariable adjusted HRs (95% CIs) in the highest quartile (≥1.07 mm) compared with the lowest (≤0.77 mm) quartile of max CCA‐IMT were 1.97 (1.26–3.06) for total stroke, 1.52 (0.67–3.41) for hemorrhagic stroke, 2.45 (1.41–4.27) for ischemic stroke, 3.60 (1.64–7.91) for lacunar infarction, 1.53 (0.69–3.41) for nonlacunar cerebral infarction, 2.68 (1.24–5.76) for CAD, and 2.11 (1.44–3.11) for CVD. The corresponding HRs (95% CIs) after further adjustment for the presence of plaque were 1.82 (1.16–2.86) for total stroke, 1.51 (0.66–3.45) for hemorrhagic stroke, 2.22 (1.26–3.89) for ischemic stroke, 3.30 (1.49–7.34) for lacunar infarction, 1.36 (0.61–3.07) for nonlacunar cerebral infarction, 2.44 (1.12–5.31) for CAD, and 1.95 (1.32–2.89) for CVD.

**Table 2:** HRs (95% CIs) of Stroke, Coronary Artery Disease, and Cardiovascular Disease According to Quartiles of Max CCA‐IMT

	Max CCA‐IMT			
	≤0.77 mm	0.78–0.95 mm	0.96–1.06 mm	≥1.07 mm
Number at risk	862	610	741	730
Total stroke				
Person years of follow‐up	12 400.95	8567.13	10 234.23	9450.57
Number of cases	36	37	45	68
Age‐, sex‐, and community‐adjusted HR	1.00	1.37 (0.86–2.17)	1.31 (0.84–2.05)	1.99 (1.31–3.03)*
Multivariable HR	1.00	1.38 (0.86–2.23)	1.38 (0.87–2.18)	1.97 (1.26–3.06)^†^
Hemorrhagic stroke				
Number of cases	14	8	9	16
Age‐, sex‐, and community‐adjusted HR	1.00	0.73 (0.30–1.75)	0.63 (0.27–1.47)	1.16 (0.55–2.45)
Multivariable HR	1.00	0.85 (0.35–2.10)	0.66 (0.27–1.65)	1.52 (0.67–3.41)
Ischemic stroke				
Number of cases	20	29	36	52
Age‐, sex‐, and community‐adjusted HR	1.00	1.97 (1.11–3.50)*	1.95 (1.12–3.38)*	2.79 (1.65–4.74)^‡^
Multivariable HR	1.00	1.87 (1.04–3.38)*	1.97 (1.12–3.45)*	2.45 (1.41–4.27)^†^
Lacunar infarction				
Number of cases	9	16	20	32
Age‐, sex‐, and community‐adjusted HR	1.00	2.33 (1.02–5.29)*	2.22 (1.00–4.90)*	3.43 (1.61–7.32)^†^
Multivariable HR	1.00	2.59 (1.13–5.96)*	2.45 (1.10–5.48)*	3.60 (1.64–7.91)^†^
Nonlacunar cerebral infarction				
No. of cases	11	13	16	20
Age‐, sex‐ and community‐adjusted HR	1.00	1.69 (0.75–3.80)	1.75 (0.80–3.80)	2.24 (1.05–4.77)*
Multivariable HR	1.00	1.30 (0.55–3.05)	1.56 (0.71–3.45)	1.53 (0.69–3.41)
Coronary artery disease				
Person‐years of follow‐up	12 550.75	8664.69	10 431.37	9679.85
Number of cases	10	19	17	31
Age‐, sex‐, and community‐adjusted HR	1.00	2.71 (1.25–5.85)*	2.01 (0.91–4.43)	3.67 (1.76–7.64)^‡^
Multivariable HR	1.00	2.54 1.17–5.52)*	1.75 (0.79–3.91)	2.68 (1.24–5.76)*
Cardiovascular disease				
Person‐years of follow‐up	12 364.28	8462.03	10 173.79	9320.50
Number of cases	45	54	60	96
Age‐, sex‐, and community‐adjusted HR	1.00	1.64 (1.10–2.44)*	1.45 (0.98–2.14)	2.35 (1.63–3.39)^‡^
Multivariable HR	1.00	1.64 (1.09–2.47)*	1.44 (0.97–2.15)	2.11 (1.44–3.11)^‡^

The multivariable adjusted HR was adjusted for age, sex, community, body mass index, systolic blood pressure, use of antihypertensive medication, diabetes mellitus, non–high‐density lipoprotein cholesterol, triglycerides, use of cholesterol‐lowering medication, eGFR, atrial fibrillation, smoking status, and drinking status. HR indicates hazard ratio; and max CCA‐IMT, maximum intima‐media thickness of the common carotid artery.

* *P*<0.05.

^†^ *P*<0.01.

^‡^ *P*<0.001.

The results according to max ICA‐IMT quartiles (Table 3) showed similar associations to those according to max CCA‐IMT, except for CAD. The multivariable adjusted HRs (95% CIs) in the highest quartile (≥1.84 mm) of max ICA‐IMT compared with the lowest (≤0.98 mm) were 1.98 (1.25–3.14) for total stroke, 1.24 (0.52–2.95) for hemorrhagic stroke, 2.45 (1.39–4.34) for ischemic stroke, 2.80 (1.24–6.32) for lacunar infarction, 2.13 (0.95–4.76) for nonlacunar cerebral infarction, 1.49 (0.74–2.98) for CAD, and 1.78 (1.20–2.62) for CVD.

**Table 3:** HRs (95% CIs) of Stroke, Coronary Artery Disease, and Cardiovascular Disease According to Quartiles of Max ICA‐IMT

	Max ICA‐IMT			
	≤0.98 mm	0.99–1.25 mm	1.26–1.83 mm	≥1.84 mm
Number at risk	735	743	758	707
Total stroke				
Person years of follow‐up	10 624.69	10 768.45	10 483.02	8776.73
Number of cases	29	40	47	70
Age‐, sex‐, and community‐adjusted HR	1.00	1.29 (0.80–2.09)	1.38 (0.86–2.20)	2.21 (1.42–3.46)^‡^
Multivariable HR	1.00	1.31 (0.81–2.14)	1.31 (0.81–2.11)	1.98 (1.25–3.14)^†^
Hemorrhagic stroke				
Number of cases	10	13	9	15
Age‐, sex‐, and community‐adjusted HR	1.00	1.22 (0.53–2.78)	0.78 (0.31–1.94)	1.41 (0.62–3.22)
Multivariable HR	1.00	1.23 (0.53–2.82)	0.64 (0.24–1.67)	1.24 (0.52–2.95)
Ischemic stroke				
Number of cases	18	26	38	55
Age‐, sex‐, and community‐adjusted HR	1.00	1.35 (0.74–2.47)	1.77 (1.01–3.13)*	2.76 (1.60–4.77)^‡^
Multivariable HR	1.00	1.37 (0.74–2.55)	1.73 (0.97–3.10)	2.45 (1.39–4.34)^†^
Lacunar infarction				
Number of cases	9	15	23	30
Age‐, sex‐, and community‐adjusted HR	1.00	1.52 (0.66–3.49)	1.98 (0.91–4.33)	2.67 (1.24–5.73)*
Multivariable HR	1.00	1.75 (0.74–4.15)	2.20 (0.97–4.97)	2.80 (1.24–6.32)*
Nonlacunar cerebral infarction				
Number of cases	9	11	15	25
Age‐, sex‐, and community‐adjusted HR	1.00	1.18 (0.49–2.86)	1.55 (0.67–3.58)	2.93 (1.33–6.41)^†^
Multivariable HR	1.00	1.04 (0.42–2.57)	1.29 (0.55–3.02)	2.13 (0.95–4.76)
Coronary artery disease				
Person‐years of follow‐up	10 778.29	10 885.14	10 563.83	9099.40
Number of cases	14	12	24	27
Age‐, sex‐, and community‐adjusted HR	1.00	0.81 (0.38–1.77)	1.55 (0.79–3.04)	1.86 (0.95–3.63)
Multivariable HR	1.00	0.79 (0.37–1.72)	1.47 (0.74–2.91)	1.49 (0.74–2.98)
Cardiovascular disease				
Person‐years of follow‐up	10 533.99	10 709.01	10 346.20	8731.40
Number of cases	42	49	71	93
Age‐, sex‐, and community‐adjusted HR	1.00	1.09 (0.72–1.65)	1.46 (0.99–2.15)	2.05 (1.41–2.99)*
Multivariable HR	1.00	1.09 (0.72–1.66)	1.39 (0.93–2.06)	1.78 (1.20–2.62)^†^

Multivariable adjusted HR was adjusted for the same variables as shown in Table 2. HR indicates hazard ratio; and max ICA‐IMT, maximum intima‐media thickness of the internal carotid artery.

* *P*<0.05.

^†^ *P*<0.01.

^‡^ *P*<0.001.

Table 4 shows the HRs (95% CIs) of total stroke, stroke subtypes, CAD, and CVD according to carotid plaque heterogeneity. The multivariable adjusted HRs (95% CIs) for subjects with heterogeneous plaques compared with those without plaques were 1.58 (1.09–2.30) for total stroke, 1.74 (1.13–2.67) for ischemic stroke, 1.84 (1.06–3.19) for lacunar infarction, 2.11 (1.20–3.70) for CAD, and 1.71 (1.25–2.35) for CVD. However, the associations were not significant for hemorrhagic stroke or nonlacunar cerebral infarction. These associations were similar to those for max CCA‐IMT.

**Table 4:** HRs (95% CIs) of Stroke, Coronary Artery Disease, and Cardiovascular Disease According to Carotid Plaque Heterogeneity

	Heterogeneity		
	Plaque (−)	Homogeneous	Heterogeneous
Number at risk	1960	487	495
Total stroke			
Person‐years of follow‐up	28 263.98	6431.2	5945.61
Number of cases	99	37	50
Age‐, sex‐, and community‐adjusted HR	1.00	1.45 (0.99–2.13)	1.86 (1.31–2.65)^‡^
Multivariable HR	1.00	1.35 (0.91–2.01)	1.58 (1.09–2.30)*
Hemorrhagic stroke			
Number of cases	29	8	10
Age‐, sex‐, and community‐adjusted HR	1.00	1.09 (0.49–2.41)	1.33 (0.63–2.81)
Multivariable HR	1.00	0.93 (0.39–2.21)	1.25 (0.58–2.70)
Ischemic stroke			
Number of cases	68	29	40
Age‐, sex‐, and community‐adjusted HR	1.00	1.65 (1.06–2.58)*	2.14 (1.42–3.21)^‡^
Multivariable HR	1.00	1.55 (0.98–2.44)	1.74 (1.13–2.67)*
Lacunar infarction			
Number of cases	39	14	24
Age‐, sex‐, and community‐adjusted HR	1.00	1.34 (0.72–2.49)	2.01 (1.18–3.41)*
Multivariable HR	1.00	1.35 (0.72–2.53)	1.84 (1.06–3.19)*
Nonlacunar cerebral infarction			
Number of cases	29	15	16
Age‐, sex‐, and community‐adjusted HR	1.00	2.11 (1.11–3.40)*	2.31 (1.22–4.37)^†^
Multivariable HR	1.00	1.82 (0.94–3.54)	1.58 (0.80–3.11)
Coronary artery disease			
Person‐years of follow‐up	28 602.24	6558.93	6153.39
Number of cases	40	12	25
Age‐, sex‐, and community‐adjusted HR	1.00	1.23 (0.64–2.37)	2.41 (1.43–4.09)^†^
Multivariable HR	1.00	1.03 (0.51–2.07)	2.11 (1.20–3.70)^†^
Cardiovascular disease			
Person‐years of follow‐up	28 047.62	6368.94	5891.94
Number of cases	135	48	72
Age‐, sex‐, and community‐adjusted HR	1.00	1.39 (0.99–1.94)	1.99 (1.47–2.68)^‡^
Multivariable HR	1.00	1.25 (0.88–1.77)	1.71 (1.25–2.35)^‡^

Multivariable adjusted HR was adjusted for the same variables as shown in Table 2. HR indicates hazard ratio.

* *P*<0.05.

^†^ *P*<0.01.

^‡^ *P*<0.001.

The corresponding HRs (95% CIs) according to plaque surface characteristics are shown in Table S1. Subjects with markedly irregular or ulcerated plaques had significantly higher HRs for CAD and CVD compared with those without plaques. The HRs (95% CIs) were 4.80 (2.18–10.55) for CAD and 2.15 (1.26–3.66) for CVD. Meanwhile, although the HRs of markedly irregular or ulcerated plaques versus no plaque were high, they were not significant for total stroke or any stroke subtype.

## Discussion

The present study showed that max CCA‐IMT was positively associated with the risks of total stroke, CAD, and CVD in the Japanese population. When we examined the associations by stroke subtypes, we observed positive associations for ischemic stroke and lacunar infarction, but not for hemorrhagic stroke and nonlacunar cerebral infarction although caution should be taken to interpret the results for hemorrhagic and nonlacunar cerebral infarction because the statistical power may be limited. We observed similar associations for max ICA‐IMT.

To the best of our knowledge, our study is the first to prospectively report on the association between CIMT and the risk of lacunar infarction in the Asian general population. Subjects in the highest quartile of max CCA‐IMT or the highest quartile of max ICA‐IMT had ≈3‐fold higher risks for lacunar infarction compared with those in the lowest quartiles. The Atherosclerosis Risk in Communities study in the United States was the only population‐based cohort study that examined the association between CIMT and incident lacunar infarction, in which the significant association was found for Black individuals, but not for White individuals.^11^

The associations of the risk of CAD with CIMT and carotid plaque in the Asian population are unclear. Several previous Western studies showed positive associations between CIMT and the risk of CAD. The Rotterdam Study, a case‐cohort study of 2073 subjects aged ≥55 years with a mean follow‐up of 4.6 years, showed that the relative risks (95% CIs) of myocardial infarction for the highest versus the lowest quartiles of maximum CIMT were 2.43 (1.38–4.27) for CCA, 4.81 (1.51–14.35) for ICA, and 3.91 (1.87–8.18) for bifurcation.^13^ The Cardiovascular Health Study, a prospective cohort study of 4476 subjects aged ≥65 years with a mean follow‐up of 6.2 years, showed that the relative risks (95% CIs) of myocardial infarction for the highest versus the lowest quintiles of maximum CIMT were 2.46 (1.51–4.01) for CCA and 3.00 (1.80–5.01) for ICA.^1^ These results in Western countries supported the association between max CCA‐IMT and CAD shown in our Japanese study, although the association between max ICA‐IMT and CAD in our study was not statistically significant, probably because of the small number of cases.

We found that heterogeneous plaque was associated with the risks of total stroke, ischemic stroke, lacunar infarction, CAD, and CVD, similar to what we found for max CCA‐IMT and max ICA‐IMT. To the best of our knowledge, no previous study has examined the association between carotid plaque characteristics and the risk of lacunar infarction. Furthermore, our study is the first to report the association between carotid plaque heterogeneity and the risk of CAD.

There are different pathophysiologic pathways of lacunar infarction.^10^, ^22^, ^23^ The major pathophysiologic pathway of lacunar infarction is lipohyalinosis, which affects intrinsic small vessels and is caused mainly by hypertension.^9^, ^24^, ^25^ The second pathway is atheroma in larger perforating arterioles or at the opening position of perforating arterioles in the parent artery (ie, middle cerebral artery). The third pathway is embolism from carotid or cardiac source, although this only accounts for no more than 10% to 15% of lacunar infarction.^26^ Because CIMT was associated with cardiovascular risk factors including hypertension and dyslipidemia,^27^ the first and second pathophysiologic pathways support the present study findings that high CIMT has an association with lacunar infarction via shared burden of risk factors. We found that the association between max CCA‐IMT and the risk of lacunar infarction remained statistically significant after further adjustment for the presence of plaque. A cross‐sectional study from the Atherosclerosis Risk in Communities Study showed that elevated CCA‐IMT was associated with the risk of lacunar infarction independently of the presence of plaque in Black subjects,^28^ which supported our results. Considering that CCA‐IMT was associated more strongly with hypertension than max ICA‐IMT and plaque,^29^, ^30^ hypertension may be considered as the main common factor relating elevated CCA‐IMT and lacunar infarction.

Heterogeneous plaque was reported to be correlated with intraplaque hemorrhage, ulceration, deposits of lipids, and cholesterol or proteinaceous material accumulation,^31^ which are features of vulnerable carotid plaques.^32^ Vulnerable carotid plaques can represent atherosclerosis, and they can be direct sources of embolism. A histopathological study showed that vulnerable plaques were associated with hypertension and dyslipidemia.^33^ Thus, the association between heterogeneous plaque and the risk of lacunar infarction in our study may be involved in all pathways of lacunar infarction.

A common cause of CAD is coronary plaque rupture, which accounts for as many as 73% of coronary thrombi.^34^ Because carotid plaque has a similar pathologic base as coronary plaque,^35^ it is likely that carotid plaque heterogeneity and surface irregularity represent coronary plaque instability and may predict the risk of CAD. Furthermore, carotid plaque surface irregularity may reflect coronary artery calcium,^36^ which increases the risk of incident CAD.^37^, ^38^ The Multi‐Ethnic Study of Atherosclerosis, a large population‐based study of 6814 participants, showed that the odds ratio (95% CI) of prevalent irregular carotid plaque surface was 1.87 (1.50–2.32) for the subjects with a positive coronary artery calcium score compared with those with a score of 0.^36^

This study has some limitations. First, we did not consider the long‐term changes in cardiovascular risk factors, CIMT, and plaque characteristics. However, it is a highly challenging problem to examine these variables repeatedly with the same accuracy, and no previous study has examined these changes. Second, we could not test interobserver and intraobserver reproducibility for ultrasound imaging. However, the reproducibility of ultrasonography findings in the present study may be comparable with those from the Cardiovascular Health Study,^16^, ^39^ because the same protocol was used and the observer (A.K.) was trained and certified in the Cardiovascular Health Study laboratory. Third, we could not calculate HRs according to CAD subtypes (myocardial infarction, angina pectoris, and sudden cardiac death) because of the small number of cases in the present study. In spite of these limitations, the strength of our study was its prospective design in the Japanese general population with long‐term follow‐up, making our findings likely to be generalizable.

In conclusion, we showed that max CCA‐IMT and heterogeneous plaque were associated with increased risks of total stroke, ischemic stroke, lacunar infarction, CAD, and CVD but not with hemorrhagic stroke and nonlacunar cerebral infarction in the Japanese general population. Max ICA‐IMT had similar associations, although it did not have a significant association with the risk of CAD. In addition, we found strong associations of markedly irregular or ulcerated plaque with CAD and CVD.

## Sources of Funding

This study was supported by grants‐in‐aid research B (11877069 from 1998 to 1999 and 12470092 from 2000 to 2001) from the Japan Society for the Promotion of Science, and the Japan Arteriosclerosis Prevention Fund for the Japan Arteriosclerosis Longitudinal Study (2001–2012).

## Disclosures

None.

## Supporting information


**Data S1**
**Table S1**
**References** **40**, **41**
