# Supplementary material for: Associations of Carotid Intima‐Media Thickness and Plaque Heterogeneity With the Risks of Stroke Subtypes and Coronary Artery Disease in the Japanese General Population: The Circulatory Risk in Communities Study
Source: J Am Heart Assoc. 2020 Sep 29;9(19):e017020. doi: 10.1161/JAHA.120.017020 (PMC7792402; doi:10.1161/JAHA.120.017020)
Supplement: Supplementary file 1 — Data S1 Table S1 References 40 , 41 [file JAH3-9-e017020-s001.pdf]

SUPPLEMENTAL MATERIAL

Data S1.

Supplemental Methods

We defined CVD as CAD and stroke. Incident CVD was ascertained in several ways. First, information on possible incidents of CVD was extracted from one of the following sources: death certificates, national health insurance claims, annual household questionnaires, annual cardiovascular risk factor surveys, and reports by local physicians, public health nurses, or health volunteers. Next, to confirm the diagnosis, we obtained clinical histories by calling, visiting, or inviting the subjects or their families to the cardiovascular risk factor surveys. Finally, we reviewed the medical records at the local clinics and hospitals. Stroke was defined, according to the criteria of the National Survey of Stroke[40], as a constellation of neurological deficits with sudden or rapid onset, persisting for at least 24 hours or until death. Stroke subtypes were primarily identified according to computed tomography (CT) or magnetic resonance imaging (MRI) findings as intraparenchymal hemorrhage, subarachnoid hemorrhage, lacunar infarction, large-artery embolism, large-artery thrombosis, unclassified large-artery infarction, or unclassified stroke[41]. CT or MRI findings were available for 91% of total stroke cases. Stroke that was diagnosed clinically but showed no lesion on CT or MRI was classified into each subtype according to clinical criteria.

The criteria for CAD were modified from those of the World Health Organization Expert Committee[42], as previously reported in detail[43]. Briefly, subjects were diagnosed with definite myocardial infarction if they had typical severe chest pain lasting ≥ 30 minutes with new abnormal and persistent Q or QS waves on electrocardiography, and/or consistent changes in cardiac enzyme levels. When typical chest pain was present, but electrocardiographic findings and enzyme levels were non-diagnostic or not obtainable, the patient was diagnosed with possible myocardial infarction. Angina pectoris was defined as repeated episodes of chest pain during effort, especially when walking, that usually disappear rapidly after the cessation of effort or the use of sublingual nitroglycerin. Sudden cardiac death was defined as death within 1 hour of symptom onset, a witnessed cardiac arrest, or abrupt collapse. CAD was defined as definite or possible myocardial infarction, angina pectoris, and sudden cardiac death.

Table S1. HRs (95% CIs) of stroke, coronary artery disease, and cardiovascular disease according to carotid plaque surface.

		Surface	
	Plaque (-)	Smooth or mildly irregular	Markedly irregular or ulcerated
No. at risk	1960	887	95
Total stroke			
Person years of follow-up	28263.98	11285.41	1091.4
No. of cases	99	78	9
Age-, sex-, and community-adjusted HR	1.00	1.66 (1.23–2.26)†	1.60 (0.80–3.20)
Multivariable HR	1.00	1.47 (1.08–2.03)*	1.43 (0.70–2.90)
Hemorrhagic stroke			
No. of cases	29	16	2
Age-, sex-, and community-adjusted HR	1.00	1.12 (0.64–2.23)	1.30 (0.30–5.63)
Multivariable HR	1.00	1.08 (0.56–2.09)	1.19 (0.27–5.24)
Ischemic stroke			

No. of cases	68	62	7
Age-, sex-, and community-adjusted HR	1.00	1.91 (1.34–2.72)‡	1.77 (0.80–3.92)
Multivariable HR	1.00	1.66 (1.15–2.39)†	1.57 (0.70–3.54)
Lacunar infarction			
No. of cases	39	32	6
Age-, sex-, and community-adjusted HR	1.00	1.61 (0.99–2.60)	2.38 (0.98–5.77)
Multivariable HR	1.00	1.54 (0.94–2.52)	2.23 (0.90–5.52)
Non-lacunar cerebral infarction			
No. of cases	29	30	1
Age-, sex-, and community-adjusted HR	1.00	2.36 (1.40–3.99)†	0.69 (0.09–5.11)
Multivariable HR	1.00	1.80 (1.04–3.13)*	0.58 (0.08–4.45)
Coronary artery disease			
Person years of follow-up	28602.24	11597.03	1115.3
No. of cases	40	28	9

Age-, sex-, and community-adjusted HR	1.00	1.55 (0.94–2.55)	4.91 (2.29–10.50)‡
Multivariable HR	1.00	1.31 (0.77–2.21)	4.80 (2.18–10.55)‡
Cardiovascular disease			
Person years of follow-up	28047.62	11191.73	1069.15
No. of cases	135	103	17
Age-, sex-, and community-adjusted HR	1.00	1.62 (1.25–2.11)‡	2.33 (1.39–3.92)†
Multivariable HR	1.00	1.42 (1.07–1.86)*	2.15 (1.26–3.66)†

*P <0.05, †P <0.01, ‡P <0.001

Multivariable adjusted HR was adjusted for the same variables as shown in Table 2.

HR: hazard ratio; CI: confidence interval
